# The role of Zic transcription factors in regulating hindbrain retinoic acid signaling

**DOI:** 10.1186/1471-213X-13-31

**Published:** 2013-08-12

**Authors:** Danna L Drummond, Caroline S Cheng, Lyndsay G Selland, Jennifer C Hocking, Lisa B Prichard, Andrew J Waskiewicz

**Affiliations:** 1 Department of Biological Sciences, University of Alberta, CW405, Edmonton, AB T6G 2E9, Canada; 2 Department of Biological Sciences, MacEwan University, Edmonton, Canada; 3 Centre for Neuroscience, University of Alberta, Edmonton, Canada; 4 Women & Children’s Health Research Institute, Edmonton, Canada

## Abstract

**Background:**

The reiterated architecture of cranial motor neurons aligns with the segmented structure of the embryonic vertebrate hindbrain. Anterior-posterior identity of cranial motor neurons depends, in part, on retinoic acid signaling levels. The early vertebrate embryo maintains a balance between retinoic acid synthetic and degradative zones on the basis of reciprocal expression domains of the retinoic acid synthesis gene *aldhehyde dehydrogenase 1a2* (*aldh1a2*) posteriorly and the oxidative gene *cytochrome p450 type 26a1* (*cyp26a1*) in the forebrain, midbrain, and anterior hindbrain.

**Results:**

This manuscript investigates the role of *zinc finger of the cerebellum* (*zic*) transcription factors in regulating levels of retinoic acid and differentiation of cranial motor neurons. Depletion of zebrafish Zic2a and Zic2b results in a strong downregulation of *aldh1a2* expression and a concomitant reduction in activity of a retinoid-dependent transgene. The vagal motor neuron phenotype caused by loss of Zic2a/2b mimics a depletion of Aldh1a2 and is rescued by exogenously supplied retinoic acid.

**Conclusion:**

Zic transcription factors function in patterning hindbrain motor neurons through their regulation of embryonic retinoic acid signaling.

## Background

During development, the vertebrate hindbrain is transiently divided into a series of lineage-restricted segments, termed rhombomeres, through the expression of distinct transcription factors. Notably, anterior-posterior patterning and segmentation of the hindbrain is critical in appropriately specifying neuronal cell types [1-5]. The identity of each hindbrain segment is regulated by the Hox family of homeobox transcription factors, the anterior expression limits of which correlate precisely with rhombomere boundaries [6-18]. The correct complement of *hox* genes expressed within each hindbrain segment specifies the identity of cells within that segment by activating regional expression of cell migration and axon guidance molecules. Blocking the functions of Hox proteins or their Pbx (Pre-B cell leukemia) and Meis (Myeloid ecotropic virus integration site) cofactors within the hindbrain leads to changes in rhombomere identity and corresponding defects in cranial motor neuron migration and axon guidance [6,17,19-21].

The vitamin A-derived morphogen retinoic acid (RA) regulates anterior-posterior patterning of the neural tube, including defining regional identity of hindbrain segments [22-28]. For example, vitamin A-deficient quail embryos lack posterior rhombomeres r4-r8 [24,25]. Maintaining the precise level of retinoic acid is critical, with increased levels known to result in teratogenic defects of the forebrain, heart, and eyes [24,29]. In the hindbrain, segmentation defects associated with changes in retinoic acid are attributed to alterations in *hox* gene expression [9,27,30-33]. For example, an increase in retinoic acid levels causes expansion of the posterior hindbrain *hox-4* expression [31,32], while a deficiency in retinoic acid causes an embryonic loss of *hox-1, hox-3,* and *hox-4* paralog expression domains [16,17,26,28].

Regional specificity of retinoic acid signaling is achieved in part through restricted domains of Retinaldehyde dehydrogenase proteins (Raldh, encoded by the *aldh1a* gene family), the enzymes that catalyze the rate-limiting step in RA synthesis [34,35]. Pharmacologic blockade of Raldh activity using diethylaminobenzaldehyde (DEAB) results in ablation of the posterior hindbrain, a phenotype that is highly analogous to the vitamin A-deficient quail [26,36]. The heme-thiolate family of cytochrome p450 type 26 enzymes (*cyp26a1/b1/c1*) hydroxylate RA, a modification that targets it for degradation [37,38]. The forebrain, midbrain and anterior hindbrain express *cyp26* genes, thereby blocking RA signaling in these regions [39-43]. The combined activity of posteriorly expressed *aldh1a* with anterior-specific *cyp26* genes creates a defined zone of RA signaling within the presumptive hindbrain. RA activity is mediated intracellularly by two nuclear receptor families, retinoid-X-receptor (RXR) and retinoic acid receptor (RAR) [44-46]. Ligand-bound heterodimeric RXR:RAR complexes activate transcription of genes containing retinoic acid response elements (RAREs). Analysis of conserved non-coding elements surrounding *hox-1* and *hox-4* paralogs has identified RAREs that are essential to rhombomere-specific expression of *hox-1*/*hox-4* genes in the hindbrain [11,31,34,47,48]. In support, alterations in RA levels result in profound defects to *hox-1* and *hox-4* gene expression domains [11,31,34,47,48].

Although the role and requirement of retinoic acid metabolism genes during embryogenesis has been extensively studied, the factors acting to initiate and maintain expression of RA metabolism genes remain largely unknown. Within vertebrates, transcription factors from the Zic (Zinc Finger of the Cerebellum) family of transcription factors are dynamically expressed in partially overlapping regions of the neural tube, indicative of a role in neural development. Recent evidence suggests a connection between Zic transcription factors and the retinoic acid signaling pathway: Maurus et al. demonstrated that loss of zebrafish Zic1 causes a decrease in presumptive forebrain expression of *cyp26a1* and an increase in RA signaling as detected by RARE:eGFP transgenics [49]. Further, mutations in human *ZIC2* result in holoprosencephaly (HPE), a forebrain defect where the cerebral hemispheres fail to separate during development [50,51] and HPE phenotypes have been connected to aberrant RA signaling, thus providing a plausible link between Zic2 and retinoic acid metabolism [29,36,52,53]. Based on these observations, we tested the hypothesis that Zic transcription factors play a key role in the initiation and maintenance of RA metabolism gene expression during zebrafish embryogenesis. The data presented here demonstrate that zebrafish *zic2* genes act upstream of retinoic acid metabolism and suggest a novel regulatory interaction between Zic2a and Zic2b transcription factors and the RA-synthesizing gene *aldh1a2*. Further, we show that Zic2 signaling is necessary for proper hindbrain patterning.

## Results and discussion

### Zic transcription factors are expressed during the initiation of RA metabolism genes

**Figure 1:**
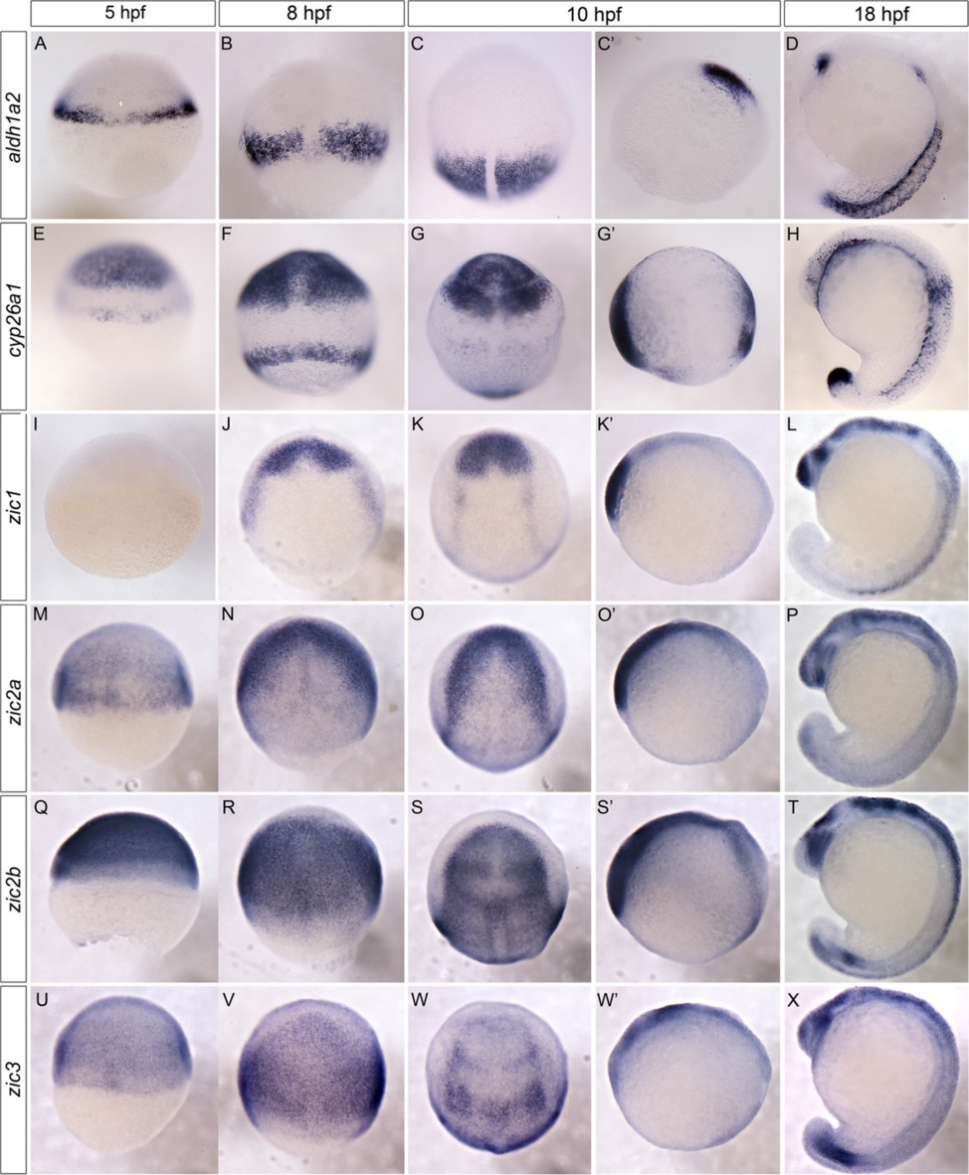
**Temporal and spatial analysis of *zic *transcription factors and key RA synthesis and degradation genes.***aldh1a2* expression is restricted to the embryonic margin at 5 hpf **(A)**. By 8-10 hpf *aldh1a2* is observed in the lateral plate mesoderm **(B, C, C’)**. At 18 hpf, *aldh1a2* is expressed in the dorsal retina and anterior somites **(D)**. *cyp26a1* is expressed in the embryonic margin and the presumptive anterior neural ectoderm at 5-8 hpf **(E, F)**. By 10-18 hpf *cyp26a1* is expressed in presumptive forebrain, midbrain, anterior hindbrain, retina, and part of the tailbud **(G, G’, H)**. *zic1* is not detectable until 8-10 hpf (75% epiboly), when it is expressed within the presumptive anterior neural tissue **(I, J, K, K’)**. By 18 hpf, *zic1* is expressed strongly in the telencephalon, midbrain-hindbrain boundary, dorsal hindbrain and spinal cord **(L)**. *zic2a* initiates earlier, with dorsally-restricted expression at 5 hpf (50% epiboly) **(M)**. By 8-10 hpf *zic2a* becomes anteriorly restricted within presumptive anterior neural tissue with additional midline expression **(N, O, O’)**. By 18 hpf, *zic2a* is within the anterior forebrain, ventral eye, dorsal hindbrain and spinal cord **(P)**. Initially, *zic2b* is expressed in a broad domain encompassing the dorsal side of the embryo (**Q**; 5 hpf). This broad expression is maintained at 8 hpf **(R)** and 10-18 hpf where expression is strongest within the eye, midbrain-hindbrain boundary, and presumptive hindbrain **(S, S’, T)**. *zic3* is also dorsally restricted at 5-8 hpf **(U, V)**. At 10-18 hpf, *zic3* is within the presumptive telencephalon, posterior forebrain, midbrain-hindbrain boundary and dorsal hindbrain, and within the tailbud **(W, W’, X)**. Images are dorsal views with anterior to top **(A-C, E-G, I-K, M-O, Q-S, U-W)** or lateral views with anterior to left **(C’, D, G’, H, K’, L, O’, P, S’, T, W’, X)**. hpf: hours post fertilization.

Retinoic acid levels are regulated by the precise action of synthesis and hydroxylation genes. Aldehyde dehydrogenase 1a2 (Aldh1a2, also known as Raldh2), the rate-limiting synthetic enzyme, catalyzes the conversion of retinal to retinoic acid [34,35,38]. The transcription of *aldh1a2* is initiated early in development, with expression at the embryonic margin at 5 hours post fertilization (hpf) (Figure 1A) and in lateral plate mesoderm by 8 hpf – 10 hpf (Figure 1B-C’) [26,54,55]. Beginning at 18 hpf, *aldh1a2* expression is restricted to the dorsal retina and anterior somites (Figure 1D). Retinoic acid degradation occurs following its hydroxylation by the cytochrome p450 oxidase, Cyp26a1. *cyp26a1* transcription is initiated early during zebrafish embryogenesis, starting with the presumptive anterior neural ectoderm at 5 hpf (Figure 1E) [56,57]. By early somitogenesis (10 hpf), *cyp26a1* mRNA is found in the presumptive forebrain, midbrain, anterior hindbrain, and part of the tailbud (Figure 1G, G’). At the later somitogenesis stages of 16-21 hpf, its expression becomes restricted to parts of the retina, caudal notochord, and tailbud (Figure 1H).

**Figure 2:**
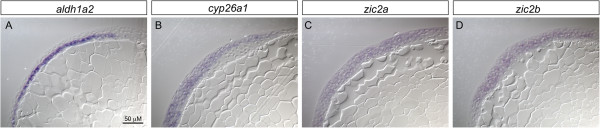
***zic2a *and *zic2b *are co-expressed with *aldh1a2 *and *cyp26a1 *in the early embryo.** 90% epiboly embryos were processed for *in situ* hybridization for *aldh1a2*, *cyp26a1, zic2a* or *zic2b*, and subsequently mounted in JB-4 resin and cut into 7 μM sections on a microtome. *aldh1a2* expression is clearly limited to the hypoblast **(A)**, while *cyp26a1* is present in the epiblast **(B)**. In contrast, *zic2a* and *zic2b* each show broad expression across the germ layers **(C,D)**.

To ascertain whether Zic transcription factors could regulate embryonic initiation of RA metabolism, we first examined which of the seven zebrafish *zics* are present during the time when *cyp26a1* and *aldh1a2* mRNA expression are initiated. Using *in situ* hybridization in conjunction with known expression data, we determined that *zic1*, *zic2a*, *zic2b*, and *zic3* are the best candidates for driving initiation of RA metabolism genes because they are expressed at the earliest stages of development [58-60]. *zic2a*, *zic2b*, and *zic3* are all expressed broadly by 5 hpf, while *zic1* expression is not detectable (Figure 1I, M, Q, U). By 8-10 hpf, *zic1* and *zic2a* are expressed in discrete regions consistent with *cyp26a1* expression within the anterior neural ectoderm (Figure 1J-K’, M-O’). *zic2b* and *zic3* show broad dorsal expression domains by 8 hpf, in regions that overlap with both anterior expression of *cyp26a1* and the more posterior expression domain of *aldh1a2* (Figure 1 R, V). As development continues, the *zics* display expression within the dorsal hindbrain, extending down the spinal cord in varying levels and degrees, showing less obvious overlap with *cyp26a1* and *aldh1a2* (Figure 1L, P, S-T, W-X). Overall, these data show that *zic2a/2b/3* transcription factor expression is consistent both temporally and spatially with the initiation of expression of *cyp26a1* and *aldh1a2.* To ensure that *zic* gene expression corresponds to the same germ layers as *cyp26a1* and *aldh1a2*, we sectioned 90% epiboly (9 hpf) embryos following *in situ* hybridization. While *cyp26a1* is expressed in the epiblast, and *aldh1a2* is limited to the hypoblast, *zic2a* and *zic2b* are present diffusely in both germ layers and are therefore co-expressed with both RA metabolism genes (Figure 2A-D).

### Zics act upstream of early retinoic acid metabolism genes, *cyp26a1* and *aldh1a2*

**Figure 3:**
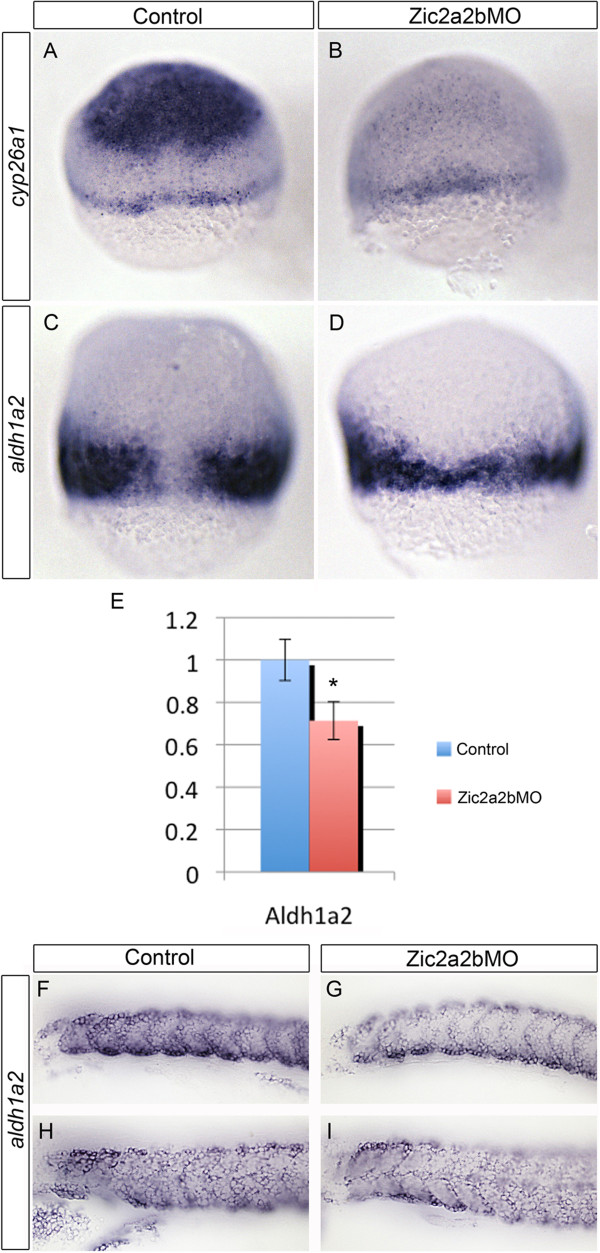
**Zic2a and Zic2b depletion causes down-regulation of the retinoid-metabolism genes, *cyp26a1 *and *aldh1a2*, during early embryogenesis.** mRNA *in situ* hybridization analysis of *cyp26a1***(A, B)** and *aldh1a2***(C, D, F-I)** expression reveals a reduction in both genes in Zic2a;Zic2b;p53 morpholino injected embryos **(B, D, G, I)** compared to control p53-morpholino injected embryos **(A, C, F, H)**. Relative *aldh1a2* mRNA expression levels for 7 hpf embryos were determined using quantitative real-time PCR **(E)**. Levels reflect average of 7 technical replicates, with *aldh1a2* mRNA at 0.71 significantly reduced (SD = 0.11; *p < 0.0001) compared to normalized control. Images are of 7 hpf embryos in dorsal view with anterior oriented to top **(A-D)**, or lateral views with anterior to the left of 18 hpf **(F, G)** or 24 hpf embryos **(H, I)**. Error bars depict standard deviation.

Based on our observations of *zic* gene expression, the earliest expressed genes *zic2a*, *zic2b*, and *zic3* were examined for possible roles in the initiation of RA metabolism*.* Due to the propensity for functional redundancy, we chose first to simultaneously knock down three transcription factors, Zic2a, Zic2b, and Zic3 with antisense morpholino oligonucleotides. Upon examining resulting phenotypes, we determined that Zic3 depletion is dispensable, and the Zic3 morpholino was removed from the injection mixture. Interestingly, embryos injected with splice-blocking morpholinos for Zic2a and Zic2b display a reduction in *cyp26a1* (Figure 3A, B) and *aldh1a2* (Figure 3C, D) expression by 7 hpf. While both the *cyp26a1* expression level and domain are reduced, the *aldh1a2* expression domain is reduced in size, but normal in intensity of staining. Results from quantitative real-time PCR showed a 29% reduction in *aldh1a2* expression in Zic2a2b-depleted embryos as compared to control embryos (p-value ≤ 0.0001, unpaired t-test) (Figure 3E). The downregulation of *aldh1a2* expression in Zic2a2b-depleted embryos can be observed as late as 18 hpf and 24 hpf (Figure 3F, G and Figure 3H, I respectively); however it is not as profound as the reduction seen in 7 hpf embryos.

### Zic depletion causes mild alterations to retinoic acid-responsive genes and hindbrain patterning

**Figure 4:**
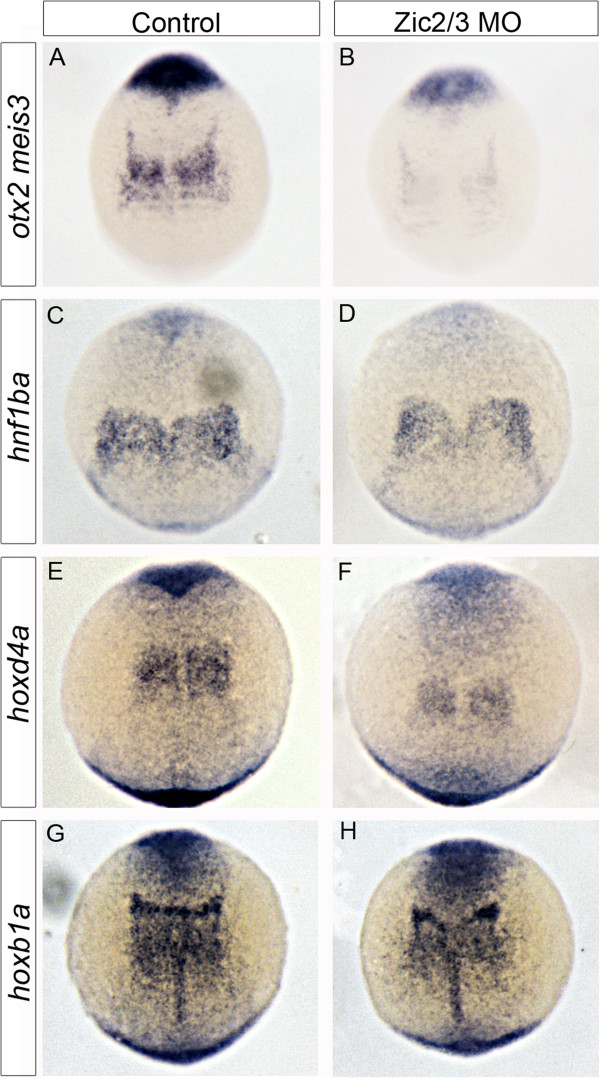
**Retinoic acid-responsive gene expression in Zic morpholino-injected embryos.** mRNA transcript expression of RA-dependent markers *meis3* and *otx2***(A, B)**, *hnf1ba***(C, D)**, *hoxd4a***(E, F)**, and *hoxb1a***(G, H)** were compared in control uninjected **(A, C, E, G)** and Zic2a;Zic2b;Zic3MO-injected embryos **(B, D, F, H)**. Although *hoxb1a* and *hnf1ba* are largely unchanged, a decrease in *meis3* and *hoxd4a* is observed in Zic morpholino-injected embryos. All images are of 10.5 hpf embryos in dorsal views with anterior oriented to top.

**Figure 5:**
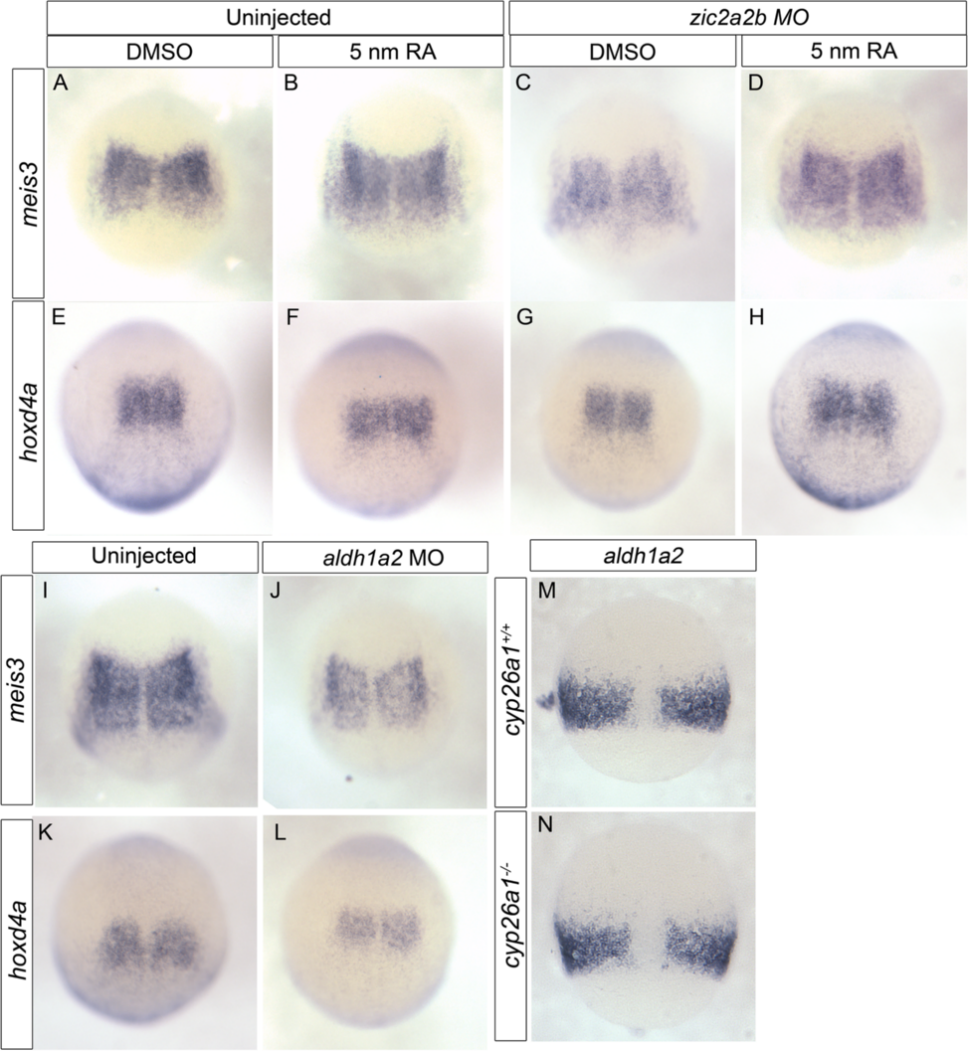
**Gene expression changes upon Zic2 knockdown can be rescued by RA treatment and is mimicked by Aldh1a2 knockdown.** mRNA *in situ* analysis of *meis3***(A-D, I-J)**, *hoxd4a***(E-H, K-L)** and *aldh1a2***(M-N)**. Uninjected embryos were treated with DMSO **(A, E)** or 5 nm RA **(B, F)** and compared to Zic2a;Zic2bMO-injected embryos treated with DMSO **(C, G)** or 5 nm RA **(D, H)**. The decrease in *meis3* and *hoxd4a* observed in Zic morpholino-injected embryos is rescued by treatment with 5 nm RA. Aldh1a2MO-injected embryos **(J, L)** show a decrease in *meis3* and *hoxd4a* when compared to controls **(I, K)**. In *cyp26a1 (giraffe)* mutants **(N)**, *aldh1a2* levels remain unchanged compared to WT siblings **(M)**. Images are dorsal views of 10.5 hpf **(A-L)** and 8 hpf **(M-N)** embryos oriented with anterior to the top.

As Aldh1a2 and Cyp26a1 have opposing effects on RA levels, the knockdown of either gene alone would be expected to produce opposite phenotypes. However, since Zic2a2b depletion reduces both RA metabolism genes simultaneously, the outcome for RA signaling is difficult to predict. Thus, we performed *in situ* hybridization for retinoic acid-responsive genes to ascertain the overall level of retinoic acid signaling in Zic-depleted embryos. Previous work demonstrated that the expression levels of zebrafish *meis3*, *hoxb1a*, *hoxd4a*, and *hnf1ba* correlate with changing retinoic acid levels (Hernandez et al., 2004, Huang et al., 2002, Kudoh et al., 2002, Moroni et al., 1993, Zhang et al., 2000). Our results show a strong reduction in *meis3* and a mild reduction in *hoxd4a* expression within the presumptive hindbrain following Zic2a2b knockdown (Figure 4A, B, E, F). Two other retinoic acid-responsive genes, *hoxb1a* and *hnf1ba,* did not show significant changes in expression with Zic2a2b morpholino when compared to uninjected controls (Figure 4C, D, G, H). To determine whether the reductions in *meis3* and *hoxd4a* are caused by changes to retinoic acid signaling, we treated *zic2a2b* morphants with 5 nM retinoic acid. Indeed, 5 nM RA is able to rescue the expression of *meis3* and *hoxd4a* to levels similar to those seen in our uninjected controls (Figure 5A-H). In addition, morpholino knockdown of *aldh1a2* causes a similar reduction in *meis3* and *hoxd4a* to that seen in *zic2a2b* morphants (Figure 5I-L). Thus, our data strongly argue that Zic2a2b promote the expression of RA-responsive genes through Aldh1a2.

We took advantage of manipulating RA levels to further examine the regulatory loops present in the early zebrafish embryo. Given that *zic2a2b* morpholinos cause a loss in *cyp26a1* expression, it is plausible that this causes an indirect effect on the *aldh1a2* domain. To test this, we examined expression of *aldh1a2* in mutants lacking *cyp26a1*. Notably, *aldh1a2* levels are unaffected in *cyp26a1(giraffe)* mutants, arguing against a scenario whereby the *aldh1a2* reduction in *zic2a2b* morphants occurs because of reduced hydroxylation of RA by Cyp26a1 (Figure 5M, N).

**Figure 6:**
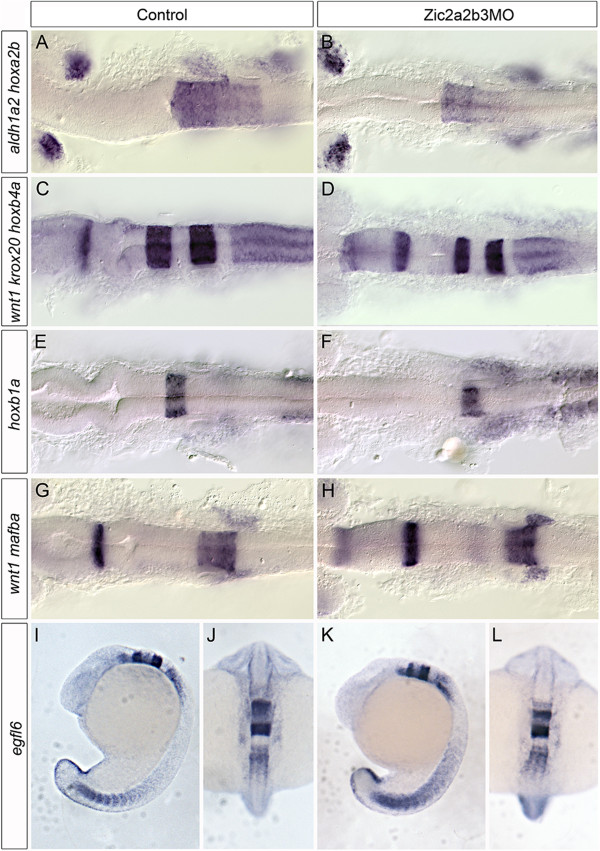
**Hindbrain patterning in Zic morpholino-injected embryos.** mRNA expression of hindbrain segmentation markers *hoxa2b/aldh1a2***(A, B)**, *krox20/hoxb4a/wnt1***(C, D)**, *hoxb1a***(E,F)**, *mafba/wnt1***(G, H)**, and *egfl6***(I-L)** were examined in control uninjected **(A, C, E, G, I, J)** and Zic2a;Zic2b;Zic3MO-injected embryos **(B, D, F, H, K, L)**. We note a reduction in *hoxa2b* expression in Zic morphant embryos (compare **B** to **A**) as well as a thinning of rhombomeres 3 and 5, as labeled by *krox20* expression (compare **D** to **C**). Expression of *hoxb4a* is reduced (compare **D** to **C**), but other markers of segment identity are overtly normal **(E-L)**. Embryos are shown in dorsal **(A-H, J, L)** or lateral **(I, K)** views and are 18 hpf.

Based on the observation that retinoic acid signaling is reduced in *zic2a2b* morphants, we examined whether *zic2a2b* depletion also led to defects in hindbrain patterning. *In situ* hybridization for hindbrain markers (*krox20, mafba, hoxb4a, hoxb1a, egfl6*) was performed to examine hindbrain segmentation and rhombomere morphology (Figure 6). We found that all rhombomeres are formed, but that rhombomeres 3 and 5 (*egr2b*/*krox20* expression) are reduced in size (Figure 6C, D) and there is reduced *hoxa2b* expression within r2-r6 (Figure 6A, B). In addition, *hoxb4a* expression within the posterior hindbrain and spinal cord is also reduced (Figure 6C, D). Other regions within the hindbrain appear normal as observed through appropriate marker expression patterns (Figure 6E-L). This suggests that although there is a reduction in the posterior neural domain, the reduction of retinoic acid signaling in *zic2a2b* morphants does not lead to a complete loss of posterior regions.

### Zic2a2b depletion reduces retinoic acid signaling

**Figure 7:**
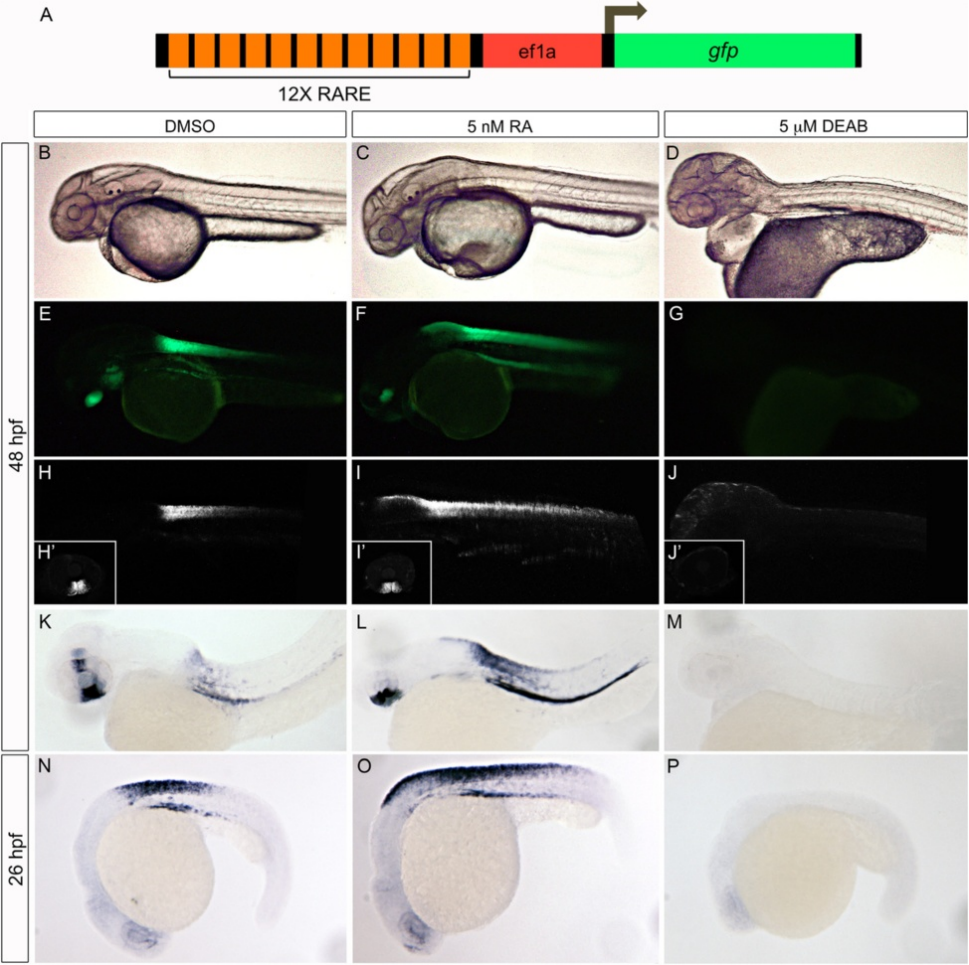
**Validation of RARE:eGFP reporter.** Tg*(12xRARE-ef1a:eGFP)*^sk71^ transgenic embryos (described in **A**) were evaluated for altered signals when RA signaling was manipulated. Embryos were treated with a vehicle control, DMSO **(B, E, H, H’, K, N)**, 5 nM all-trans retinoic acid **(C, F, I, I’, L, O)**, or 5 μM DEAB **(D, G, J, J’, M, P)**. We note an expansion of hindbrain/spinal cord expression of the transgene in 5 nM RA-treated embryos, and a complete loss in 5 μM DEAB-treated embryos. Altered expression is detectable by examination of fluorescence using a stereomicroscope **(B-G)**, confocal microscope **(H-J’)**, or by *in situ* hybridization with a probe to *eGFP***(K-P)**. All embryos are shown in lateral view at 48 hpf **(B-M)** or 26 hpf **(N-P)**. The inset panels **(H’-J’)** display reporter activity in the ventral retina.

The transgenic zebrafish line Tg*(12xRARE-ef1a:eGFP)*^sk71^ contains twelve retinoic acid response elements (RAREs) upstream of a ubiquitous promoter linked to the gene for enhanced green fluorescent protein, *eGFP*[61,62]. Active retinoic acid signaling in Tg*(12xRARE-ef1a:eGFP)*^sk71^ fish can be detected either directly by observation of eGFP fluorescence, or by *in situ* hybridization for *eGFP* mRNA. The sensitivity of this transgenic line was examined by assaying its response to alterations in retinoic acid levels (Figure 7). In 26 and 48 hpf control embryos, the predominant regions of retinoic acid signaling are within the spinal cord and posterior hindbrain (Figure 7B, E, H, K, N). As expected, the fluorescence domain is increased at both stages in response to retinoic acid treatment, expanding further anteriorly into the hindbrain and posteriorly down the spinal cord (Figure 7C, F, I, L, O). At 48 hpf, there are additional retinoic acid signaling centers within the dorsal and ventral eye (Figure 7H’, K). Here, exogenous addition of retinoic acid results in morphologically smaller eyes and loss of the dorsal expression domain, while the ventral *eGFP*-positive region is maintained (Figure 7I’, L). Blocking retinoic acid synthesis with DEAB treatment results in a loss of all retinoic acid signaling, with no fluorescence or *in situ* coloration visible at 26 hpf or 48 hpf (Figure 7D, G, J, J’, M, P). These results demonstrate that the Tg*(12xRARE-ef1a:eGFP)*^sk71^ zebrafish line is a suitable tool for studying retinoic acid signaling. Of note, we were unable to detect the transgene before 24 hpf, preventing the analysis of retinoic acid signaling levels at early embryonic stages, when retinoic acid metabolism genes are first transcribed.

**Figure 8:**
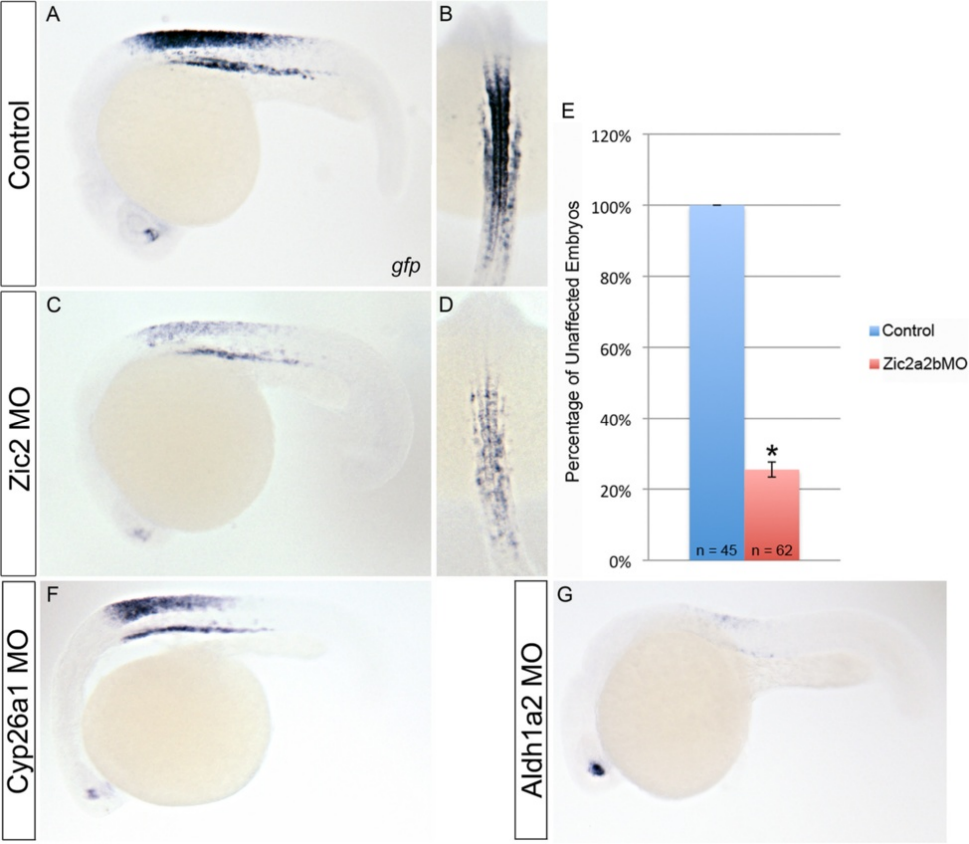
**Zic2a and Zic2b depletion reduces RA signaling levels.** Tg*(12xRARE-ef1a:eGFP)*^sk71^ transgenic embryos were used to assay the level of RA signaling. To increase sensitivity of transgene detection, *in situ* hybridization for *eGFP* was employed. Control p53MO-injected embryos **(A, B)** were compared with Zic2a;2b;p53MO-injected embryos **(C, D)**. Quantification of reduction in *eGFP* in Zic2a;2b;p53 morphants is displayed graphically with reduced expression in 75 ± 2% of embryos (p-value = 0.0004; unpaired t-test). Cyp26a1 **(F)** and Aldh1a2 **(G)** morpholinos were also injected into *Tg(12xRARE-ef1a:eGFP)*^sk71^ embryos to compare effects with those obtained using Zic morpholinos. Images are of 26 hpf embryos in lateral **(A, C, F, G)** and dorsal **(B, D)** views with anterior to left and top, respectively.

As the Tg(*12xRARE-ef1a:eGFP*) transgenic line labels posterior retinoic acid signaling regions, we asked whether the reduction in retinoic acid metabolism genes following Zic depletion leads to an alteration in later embryonic retinoic acid signaling levels. As compared to control *eGFP* levels (Figure 8A, B), there is a strong reduction in *eGFP* expression in 75% of the *zic2a2b* morphant embryos at 26 hpf (Figure 8C, D, E). This suggests that there is a reduction in retinoic acid signaling within the Zic2a2b-depleted hindbrain at later stages. To test the hypothesis that the reduction in retinoic acid signaling in *zic2a2b* morphants is a result of reduced *aldh1a2* expression, we compared retinoic acid signaling between Aldh1a2-depleted and Cyp26a1-depleted embryos. *eGFP* levels appear only slightly affected in *cyp26a1* MO-injected embryos as compared to control (Figure 8A, F). However, strikingly, there is a strong reduction in retinoic acid signaling levels across both Zic2a2b and Aldh1a2 knockdowns, as shown by strong reduction of *eGFP* transcript as compared to controls (Figure 8A, C, G). Taken together, these data show that we have identified a novel regulatory mechanism whereby Zic2a2b regulates retinoic acid levels, most likely through the regulation of *aldh1a2* transcription.

### Vagal neurons are sensitive to alterations in retinoic acid levels

**Figure 9:**
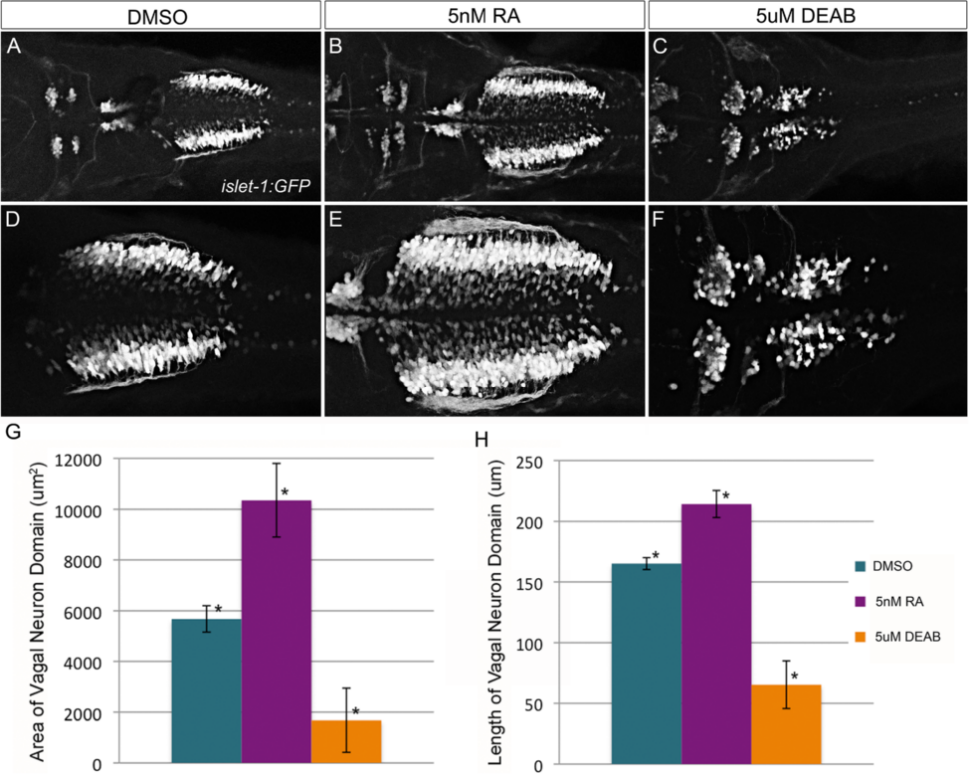
**Vagal neurons are sensitive to alterations in retinoic acid levels.***Tg(isl1:eGFP)* transgenic zebrafish are used to visualize alterations to branchiomotor neurons in response to 1% DMSO **(A, D)**, 5 nM all-trans retinoic acid **(B, E)**, or 5 μM DEAB **(C, F)** treatment. Images are dorsal views of 48 hpf embryo hindbrain using 20× or **(A-C)** or 40× **(D-F)** objective. Length **(G)** and area **(H)** were quantified using ImageJ and displayed graphically (error bar denotes standard deviation). The differences induced by either retinoic acid or DEAB treatment are statistically significant (ANOVA and Tukey’s HSD post-hoc test, *p-value < 0.01).

Our results demonstrated strong deficits in RA signaling at 26 hpf, a key time-point for specification and differentiation of hindbrain branchiomotor neurons [1]. To determine the developmental consequence of the reduction in retinoic acid signaling, we chose to examine development of vagal motor neurons within the posterior hindbrain and spinal cord [55]. Using the *Tg*(*isl1:eGFP*) transgenic line, we sought to analyze development of hindbrain branchiomotor neurons by confocal microscopy in response to Zic2a2b depletion [63]. In control embryos, trigeminal neuron cell bodies are located within rhombomeres 2 and 3, facial neurons within rhombomeres 5 and 6, and the vagal neuron domain extends from the posterior hindbrain down the spinal cord (Figure 9A, D). To estimate the number of vagal neurons, we used ImageJ to quantify the area and length of the vagal neuron cluster (Figure 9G, H). Upon treatment with retinoic acid, the vagal domain is greatly expanded and more neuronal cell bodies are visible as compared to control (Figure 9B, E, G, H). The average area in DMSO-treated control embryos is 5481 μm^2^ (SD = 321 μm^2^) and the anterior-posterior length is 165 μm (SD = 4.5 μm). Treatment with RA results in an average area of 10,353 μm^2^ (SD = 1448 μm^2^; p-value ≤ 0.01) and length of 214 μm (SD = 11 μm; p-value ≤ 0.01). Alternatively, treatment with DEAB, a pharmacological inhibitor of retinoic acid synthesis, significantly alters embryonic patterning such that all posterior rhombomeres are lost. The vagal domain is almost undetectable in these embryos (Figure 9C, F, G, H). DEAB treatment also results in mispatterning of anterior branchiomotor neuron classes such that it is difficult to identify trigeminal, facial, and possible remnants of vagal neuron populations. As a best estimate, the area of vagal neurons in DEAB-treated embryos is 1683 μm^2^ (SD = 1264 μm^2^; p-value ≤ 0.01) and length is 65 μm (SD = 20 μm; p-value ≤0.01).

### Zic2a2b knockdown causes loss of vagal neurons

**Figure 10:**
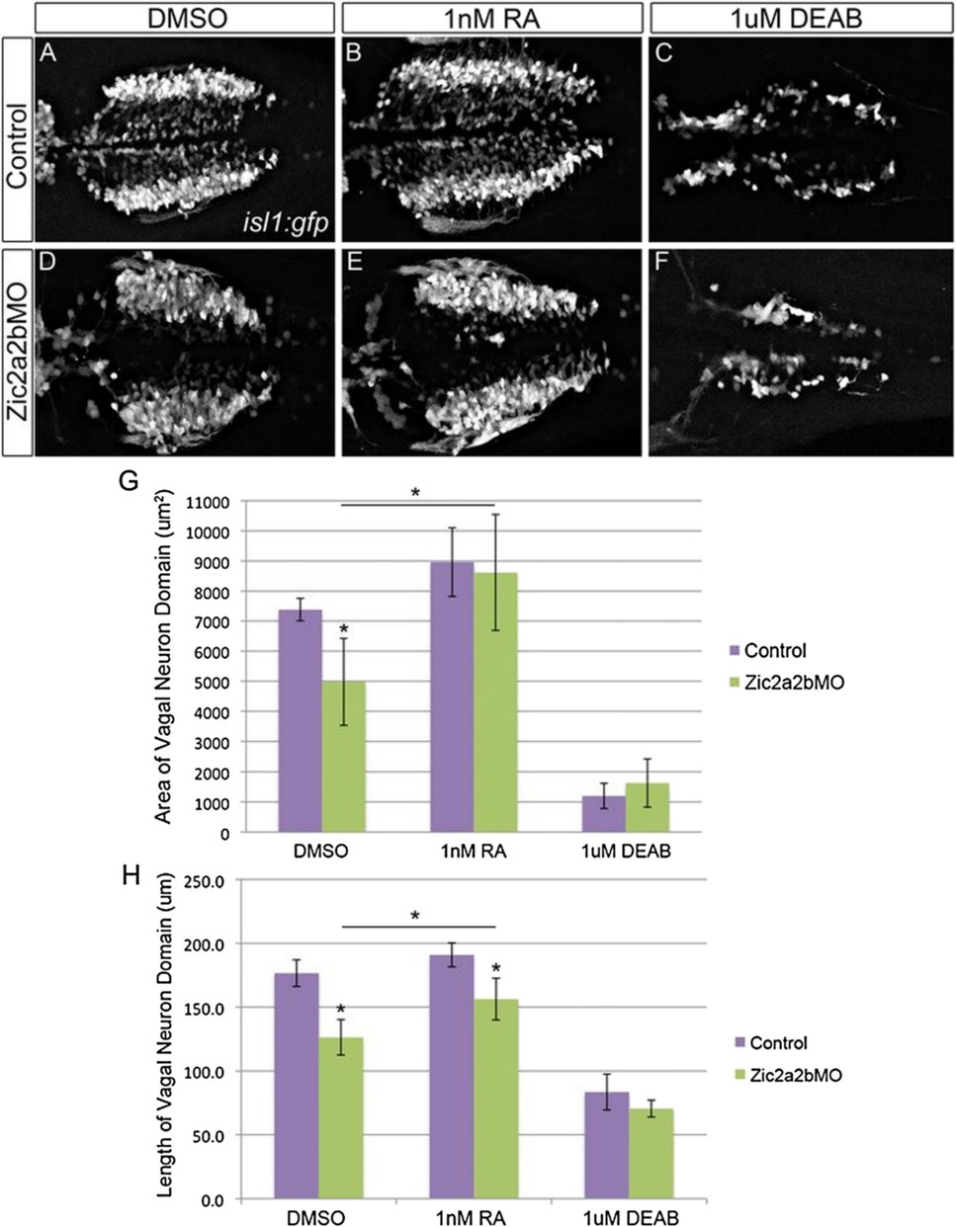
**Vagal neuron domain in Zic2a and Zic2b morpholino injected embryos is partially rescued with low doses of RA.** Dorsal views of the vagal motor neurons in 48 hpf Tg(*isl1:eGFP*) embryos that were injected with p53 morpholino **(A-C)** or Zic2a;Zic2b and p53 morpholinos **(D-F)**, and treated with 1% DMSO **(A, D)**, 1 nM RA **(B, E)**, or 1 μM DEAB **(C, F)**. Quantification of vagal motor neuron domain area **(G)** demonstrates that Zic2a/2b depletion causes a statistically significant reduction in domain size, which is rescued by treatment with retinoic acid. Quantification of vagal neuron domain length **(H)** gives similar results, with a statistically significant reduction upon Zic2 depletion, which could again be counteracted by retinoic acid addition. Statistical significance calculated by ANOVA and Tukey’s HSD post-hoc test (*p-value ≤ 0.01).

Zic2a2b-depleted embryos have reduced retinoic acid signaling levels within the posterior hindbrain and spinal cord. To ascertain whether this reduction in retinoic acid signaling causes a concomitant defect in vagal motor neurons, Zic2a2b knockdown was introduced in Tg(*isl1:eGFP*) transgenic zebrafish. Embryos were grown in media containing DMSO (control) or treated with exogenous retinoic acid (1 nM) or DEAB (1 μM) to determine if retinoic acid supplementation could rescue phenotypes. Zic-depleted embryos have significantly reduced vagal neuron area and length as compared to control embryos (Figure 10A, D, G, H). The average area of the vagal neuron domain in Zic-depleted embryos is 4979 μm^2^ (SD = 1443 μm^2^) as compared to 7382.5 μm^2^ (SD = 377.2) in controls. The average length of the vagal neuron domain is 126.4 μm (SD = 13.8) as compared to 176.6 μm (SD = 10.5) in controls (n = 15, p-value < 0.01). This reduction in the vagal neuron domain would be expected with a mild reduction in retinoic acid levels. In many cases, the medial-lateral width of each vagal population was increased in morphants, suggesting a possible migrational error, as vagal neurons are typically formed medially and migrate laterally during development [1]. The physiological level of retinoic acid is estimated to be 3 nM [64,65]. Due to reduced retinoic levels in Zic-depleted embryos, we postulated that supplementation with near physiological levels (1 nM) of retinoic acid (a concentration that does not cause significant alteration to control embryos) may be sufficient to rescue the neural phenotype seen. There is a mild increase in vagal neuron area and length in control embryos in response to 1 nM retinoic acid treatment (Figure 10B, G, H). Strikingly, with low dose treatment of retinoic acid, the vagal neuron area of Zic-depleted embryos is rescued to values similar to those seen in control embryos: RA-treated, Zic-depleted embryos have an average vagal neuron area of of 8616.4 μm^2^ (SD = 1929 μm^2^) as compared to an average area in control embryos of 8963.8 μm^2^ (SD = 1139.1 μm^2^) or 4979.3 μm^2^ (SD = 1443 μm^2^) in DMSO-treated *zic2a2b* morphants. There is also a significant increase in the length of the vagal domain in *zic2a2b* morphants treated with retinoic acid as compared to morphants in DMSO (vehicle control) conditions (Figure 10D, E, G, H): RA-treated morphants have a vagal domain length of 156 μm (SD = 16 μm), compared to 126 μm (SD = 14; p-value < 0.01; n = 14) for DMSO-treated morphants. While the domain length after RA treatment is similar to that of untreated control embryos, it is still significantly reduced compared to control embryos treated with retinoic acid. We asked whether zic2a2b morphants had residual RA by treating them with DEAB. By further reducing retinoic acid levels with DEAB treatment, there is a roughly equivalent shrinkage of the vagal neuron domain in both *zic2a2b* morphants and control embryos (Figure 10C, F, G, H).

**Figure 11:**
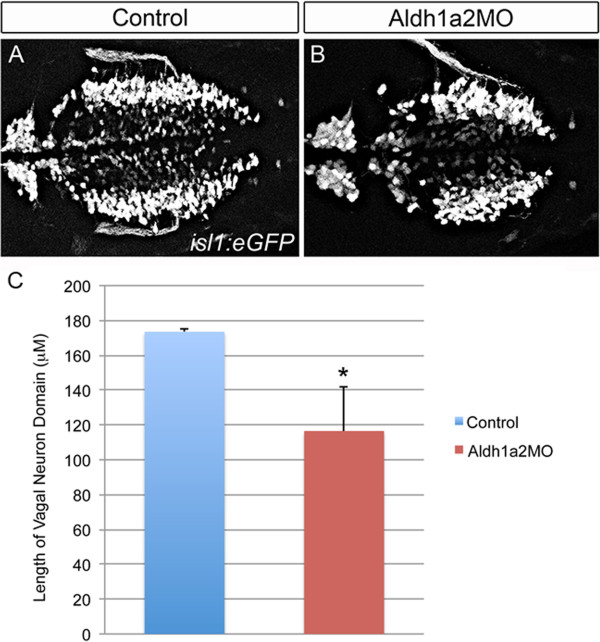
**Knockdown of Aldh1a2 causes a reduction in the vagal neuron domain.** Analysis of vagal motor neurons in Tg(*isl1:eGFP*) transgenic embryos injected with either a p53 control morpholino (1 ng, **A**) or an Aldh1a2 morpholino (2 ng, **B**). The length of the vagal domain was quantified using Image J **(C)**. Significance calculated using an unpaired t-test (*p-value ≤0.01; n = 2, 6 for quantification of p53MO and Aldh1a2 MO, respectively).

Our data is consistent with a model whereby Zic2a and Zic2b act upstream of retinoic acid metabolism by activating transcription of *aldh1a2*. As shown, there is an early reduction in *aldh1a2* transcript levels in Zic2a2b morphants, and the reduction in retinoic acid signaling levels within Tg(*12xRARE-ef1a:eGFP*) transgenics is similar in Zic2a2b and Aldh1a2-depleted embryos. Further, the reduction in vagal neurons in *zic2a2b* morphants is rescued by treatment with retinoic acid, the product of an Aldh1a2-catalyzed reaction. To determine if this relationship between Zic2a2b and *aldh1a2* is maintained in later neurons, vagal neurons were examined in *aldh1a2* morphant Tg(*isl1:eGFP*) zebrafish. The Aldh1a2-depleted embryos also show a statistically significant reduction in length of the vagal domain, very similar to that seen in Zic2a2b-depleted embryos (compare Figures 10 and 11). The comparable reduction in vagal domain length supports the hypothesis that Zic2a2b act upstream of *aldh1a2*. Taken together, our data show that the most posterior class of branchiomotor neurons, the vagal neurons, is sensitive to alterations in retinoic acid levels. Increased retinoic acid causes an expansion in the area and length of the vagal neuronal population, while retinoic acid depletion reduces the population. Consistent with this observation, the reduction in retinoic acid signaling levels in Zic2a2b-depleted embryos elicits a reduction in the size of the vagal neuron domain. Importantly, the vagal neuron domain in Zic2a2b-depleted embryos can be rescued by supplementation of RA to physiological levels.

## Conclusions

We have identified a novel regulatory mechanism between Zic2a2b transcription factors and early retinoic acid signaling levels. We propose a model whereby Zic2a and Zic2b act upstream of both retinoic acid synthesis (*aldh1a2*) and degradation (*cyp26a1*) genes in an early embryo. We see a persistent reduction in retinoic acid signaling in Zic2a2b morphants at 26 hpf that is similar to that observed in embryos depleted of the main retinoic acid synthesis enzyme, Aldh1a2. Consistent with reduced retinoic acid levels, Zic2a2b knockdown results in reduced vagal motor neuron formation within the posterior hindbrain and spinal cord. This phenotype is nearly identical to Aldh1a2-depleted embryos and is rescued by exogenous retinoic acid treatment.

While the role and requirement for retinoic acid metabolism genes has been well studied, less is known about the upstream regulators of these factors. During mouse development, Hoxa1-Pbx1 (Pre-B-cell leukemia homeobox 1) complexes directly regulate Raldh2 transcription within mesodermal tissue [66]. Additionally, deficiencies in zebrafish Tgif and Hmx4 cause defects associated with reduced retinoic acid levels and reduced *aldh1a2* transcription [36,67]. Interestingly, Tgif-depletion also results in a concomitant reduction in *cyp26a1* mRNA levels, similar to defects observed in Zic-depleted embryos. As expected, the *Cyp26a1* murine mutant has defects associated with increased retinoic acid levels [41]. More notably, rescue of these defects is observed with heterozygous disruption of *Aldh1a2* in a *Cyp26a1* murine mutant, suggesting co-dependency between these genes, where less retinoic acid degradation is necessary when retinoic acid synthesis is reduced [38]. Thus, the fact that the transcription factors Zic2a and Zic2b regulate expression of both retinoic acid and synthesis genes strongly suggests that they play an important role in regulating retinoic acid levels during early embryogenesis.

Holoprosencephaly (HPE) is the most common forebrain birth defect, occurring when the cerebral hemispheres fail to separate. Mutations in human *ZIC2* and *TGIF* are known to cause HPE. Our results from zebrafish studies provide evidence that both of these genes function to regulate RA metabolism [36]. Although both *Zic* and *Tgif* genes are implicated in regulating multiple signaling pathways, it is now plausible that altered levels of RA predispose embryos to HPE.

## Methods

### Zebrafish care

*Danio rerio* were maintained in accordance with published protocols [68]. Zebrafish adults and embryos were maintained at 28.5°C. Embryos were raised in embryo medium (EM) with 10 ml/l Penicillin/Streptomycin (Sigma) added to prevent bacterial growth, and with phenyl-thiourea (0.003%) used to prevent pigmentation after 24 hpf. The stage of developing embryos was assessed using reported morphological guidelines (Kimmel et al., 1995). Wild type AB, Tg(*isl1:eGFP*), and Tg(*12xRARE-ef1*μ:eGFP) strains were used as described [61-63]. Animal protocols were approved by the University of Alberta’s Animal Care and Use Committee-Biosciences with protocol #427.

### Morpholino injections

Splice-blocking Zic2aMO [ 2 ng/ml; 5’-CTCACCTGAGAAGGAAAACATCATA-3’; [69]], splice-blocking Zic2bMO [2 ng/ml; 5’-CACGAATTGAAATAATTACCAGTGT-3’], splice-blocking Zic3MO [3 ng/ml; 5’-GGAATTTAATTTCCTTACCTGTGTG-3’], translation-blocking Aldh1a2MO [2 ng/ml; 5’-GCAGTTCAACTTCACTGGAGGTCAT-3’; [54,70]], translation-blocking Cyp26a1MO [2 ng/ml; 5’-CGCGCAACTGATCGCCAAAACGAAA-3’; [71]], and p53MO morpholinos [1 ng/ml] were designed to produce a non-functional protein [72]. Embryos were injected at the one-cell stage and allowed to develop until the desired time-point for phenotypic characterization.

### Examination of retinoic acid signaling

To manipulate retinoic acid levels in developing zebrafish embryos, we used pharmacological treatment (1 or 5 nM of all-trans retinoic acid (Sigma); 1 or 5 μM diethylaminobenzaldehyde (DEAB, Sigma)) or morpholinos (Cyp26a1 or Aldh1a2) that target enzymes known to regulate degradation and synthesis of endogenous RA. Embryos were treated at approximately 50% epiboly with DEAB or RA in Embryo Medium, with DMSO and ethanol used as vehicle controls. Embryos were manually dechorionated at 26 hpf and, maintaining treatment concentration, media was changed once per day. All treatments were carried out in 60 mm petri dishes each containing approximately 40 embryos grown at 28.5°C. Tg(*12xRARE- ef1a:eGFP*) transgenic zebrafish allow visualization of RA-signaling levels by assaying fluorescence (or by *in situ* hybridization for *eGFP* mRNA).

### Whole mount *in situ* hybridization

mRNA *in situ* hybridization procedure is based on previously published methods [36]. Probes were synthesized via a PCR-based approach whereby primers are designed to amplify the 3’ untranslated region (primer sequences available on request) [73]. Embryos of desired stage were fixed in 4% paraformaldehyde (PFA), permeabilized in Proteinase K (10 μg/ml), re-fixed in 4% PFA and pre-hybridized for 2 hours at 65°C. 100 μg of probe was added and hybridization allowed to proceed overnight. Unbound probe was removed with three 20-minute high-stringency washes (0.2× SSC + 0.1% Tween-20; 0.1× SSC + 0.1% Tween-20; 0.1× SSC + 0.1% Tween-20). Embryos were first incubated in Blocking Solution (2% Sheep Serum + 2 mg/ml Bovine Serum Albumin in PBST) for one hour at room temperature, or 4°C overnight, before transfer into primary antibody (Blocking Solution + 1/5000 dilution of sheep anti-DIG-AP-FAB fragments antibody (Roche)) for two hours at room temperature, or 4°C overnight. Embryos are washed out of antibody using five-fifteen minute PBST washes. The coloration reaction is performed with either nitro-blue tetrazolium (NBT)/bromo-4-chloro-3-indolyl phosphate (BCIP) stock (Roche) in Coloration Buffer or, for embryos before bud stage, with BM purple (Roche). Coloration was stopped via 100% methanol/0.1% Tween-20 washes. Deyolked embryos were dehydrated in 50% glycerol, then 70% glycerol before mounting. Images are taken on Zeiss Axio Imager.Z1 using Axovision SE64 Rel.4.8 software. Embryos in early development (6-14 hpf) or with yolk attached are photographed on Olympus SZX12 stereoscope using QImaging micropublisher camera.

### JB-4 sectioning

After *in situ* hybridization, embryos were post-fixed for 2 hours at room temperature in 4% paraformaldehyde. Embryos were then rinsed in PBS, dehydrated through a series of ethanol washes, and incubated over one hour in two rinses of JB-4 infiltration solution (made as per manufacturer’s instructions, Polysciences Inc.). After transferring the embryos to molds, the infiltration solution was replaced with JB-4 embedding media and the blocks were left to harden at room temperature overnight. Seven μM sections were cut on a Leica RM2235 microtome and images were captured on a Zeiss Axio Imager.Z1 using Axovision SE64 Rel.4.8 software.

### Quantitative real-time PCR

Quantitative Real-Time PCR was used to quantify *in vivo* mRNA levels to ascertain *aldh1a2* gene expression in morpholino-injected embryos. Primers were designed using Roche Universal Probe Library for Zebrafish [Forward 5’-AACCACTGAACACGGACCTC-3’ and Reverse 5’-ATGAGCTCCAGCACACGTC-3’]. After RNA isolation (Ambion RNAqueous), DNA was removed by DNAseI digestion (19 μl DEPC-treated H_2_O, 10 μl 10× DNAseI buffer, 1 μl DNAseI (Ambion)) for 30 minutes at 37°C. RNA was further purified using Qiagen RNeasy columns and cDNA synthesized using AffinityScript qPCR cDNA Synthesis kit (Ambion) by the recommended protocol. Quantitative RT-PCR and primer validation was carried out as previously described [74,75]. For primer validation, control (AB) cDNA was isolated and used to create a dilution series: 1/8, 1/16, 1/32, 1/64, 1/128, 1/256. Ambion Brilliant II qPCR kit with thermocycler conditions 40× denaturation at 95°C for 30 seconds; annealing at 55°C for 1 minute; and extension at 72°C for 30 seconds. Finally, relative gene expression level was determined using the comparative Ct method (2^−∆∆Ct^ method) and unpaired t-test calculations for significance [74,75]. Reaction mixtures and thermocycler program were identical to primer validation protocol; a 1/16 dilution of experimental and control cDNA was used with each primer pair. Reactions were completed with at least 7 technical replicates from RNA isolated from 50 embryos, comparing to *ef1a* as endogenous control.

### Imaging of cranial motor neurons

The Tg(*isl1:eGFP*) transgenic line allows visualization of a subset of motor neurons including those that are located within midbrain and hindbrain: oculomotor, trochlear, trigeminal, facial, glossopharyngeal, and vagal neurons [63]. For fluorescent analysis, embryos were fixed with 4% PFA for 4 hours at room temperature. Yolks were removed manually and embryos dehydrated in 50% and 70% glycerol before mounting and imaging using a Zeiss LSM 510 confocal microscope and Zeiss Zen software. Image preparation and analysis (quantification of vagal neuron fluorescent area and length of vagal domain) was done with ImageJ software. To calculate vagal area, Z-stacked images were converted to 8-bit color and scale was set to correspond with that of image (pixel length 1024 or 2048, length of field of view 319.93 μm). Image threshold was set capturing the greatest number of neurons while keeping noise low. Regions of fluorescence were selected and the area calculated with numerical information transferred to Microsoft excel, where totals and averages were calculated. Statistical significance was determined using ANOVA followed by Tukey’s HSD post hoc test, or by an unpaired t-test.

## Competing interests

The authors declare that they have no competing interests.

## Authors’ contributions

DLD conducted the studies of *zic* expression, morpholino knockdown, and analysis of retinoic acid signaling. CSC conducted studies of *zic* expression and assisted with writing the manuscript. LGS assisted with writing the manuscript and performed control experiments. JCH performed the JB-4 sectioning, helped with data analysis and edited the manuscript. LBP conducted studies of Zic function in regulating ocular retinoic acid levels and wrote the manuscript. AJW conceived of the study, participated in its design and wrote portions of the manuscript. All authors read and approved the final manuscript.
